# Enhancement of nutritional and flavor properties of red-fleshed pitaya juice via *Lacticaseibacillus paracasei* fermentation

**DOI:** 10.1016/j.fochx.2025.102915

**Published:** 2025-08-13

**Authors:** Jiayu Gu, Qian Zhu, Jingjing Du, Jiagang Guo, Yuhan Wu, Song Yang, Jian Jiang

**Affiliations:** a Institute of Agro-products Processing, Anhui Academy of Agricultural Sciences, Hefei, Anhui 230031, China; b Anhui Engineering Laboratory for Functional Microorganisms and Fermented Foods, Hefei, Anhui 230031, China

**Keywords:** Red-fleshed pitaya juice, *Lacticaseibacillus paracasei*, Fermentation, Volatile compounds, Antioxidant activity

## Abstract

In this study, the changes in physicochemical properties, antioxidant activity, and volatile/non-volatile metabolites of red-fleshed pitaya (*Hylocereus polyrhizus*) juice during *Lacticaseibacillus paracasei* fermentation were comprehensively investigated. The results indicated that *L. paracasei* significantly increased the total polyphenols, flavonoids, and anthocyanins contents, with the total phenolic content increasing from 0.16 ± 0.03 mg/mL to 0.38 ± 0.03 mg/mL. *L. paracasei* fermentation enhanced the antioxidant activities, lipase inhibition, and α-glucosidase activities of red-fleshed pitaya juices. A total of 78 volatile compounds were detected. The increased abundance of volatile compounds significantly enhanced the flavor profile, adding floral, fruity, buttery, and sweet flavors. Furthermore, metabolomic analysis demonstrated that *L. paracasei* significantly altered the non-volatile compounds of red-fleshed pitaya juice, particularly increasing ornithine, asparagine, glutamine, and indole-3-lactic acid. These metabolites are involved in carbohydrate metabolism, amino acid synthesis/metabolism pathways, suggesting their potential roles in nutritional improvement. These improvements demonstrated its potential as a functional beverage.

## Highlights

• *Lacticaseibacillus paracasei* was used for red-fleshed pitaya juice fermentation.
• Fermentation increased bioactive compound contents of pitaya juice.
• Fermentation improved antioxidant activity of red-fleshed pitaya juice.
• Fermentation improved lipase and glucosidase inhibition ability of juice.
• Fermentation increased content of volatile/non-volatile compounds.

## 1 Introduction

Red-fleshed pitaya (*Hylocereus polyrhizus*) is an important tropical fruit that is native to Mexico and Central and South America. Recently, the cultivation and consumption red-fleshed pitaya has gradually spread worldwide to tropical and subtropical countries such as China due to its promising nutritional and antioxidant properties (Zheng et al., 2025). Red-fleshed pitaya is a rich source of diverse bioactive compounds, notably betacyanins, phenolic compounds, flavonoids, and terpenoids. Emerging evidence suggests that pitaya consumption may confer various potential health benefits (Shah et al., 2023). Ravichandran et al. (2020) highlighted red-fleshed pitaya as a promising natural source of anti-glycation and antioxidant compounds, potentially mitigating glycation-related risks in diabetes and aging-related complications, with phenolic compounds such as 4- prenylresveratrol, vicenin being the key contributors through *in vitro* and molecular docking experiments. Therefore, red-fleshed pitaya has the potential to serve as a source of functional ingredients that provide nutrients to health-promoting (Jiang et al., 2021).

Consumption of red-fleshed pitaya is generally limited to fresh fruit and fruit juice. With consumer preferences shifting toward higher-quality and less-processed juices, probiotic fermentation is receiving attention from the scientific world as well as consumers, as evidenced by recent studies focusing on fruit juice fermentation (Gupta & Abu-Ghannam, 2012). Lactic acid bacteria (LAB) fermentation of fruits is a sustainable process that aims to retain the sensory and nutritional features of the raw matrices and extend shelf life under safe conditions (Filannino et al., 2014). LAB enhance the flavor and food quality of the fermented substrate mainly due to carbohydrate metabolism, proteolysis and amino acid metabolism, lipolysis and fatty acid metabolism. Lactic acid bacteria such as *Lactiplantibacillus plantarum*, *Lacticaseibacillus casei*, *Lacticaseibacillus rhamnosus*, and *Leuconostoc mesenteroides* are candidates for juice fermentation (Filannino et al., 2014; Yang et al., 2024). Zhang et al. (2021) reported that fermentation with *L. plantarum* J26 enhanced functional compounds in fruit juices, as demonstrated in blueberry juice study. Although blueberry and pitaya possess distinct phytochemical profile, this suggests the broad potential of LAB fermentation for functional juice development. Our study extends LAB fermentation to red-fleshed pitaya juice to improve its flavor and nutrition.

Red-fleshed pitaya juice possesses physicochemical characteristics suitable for probiotic cultivation, including optimal pH, fermentable sugars and prebiotic fibers that may enhance probiotic viability and metabolic activity (Gengatharan et al., 2021). *Lactobacillus acidophilus* FNCC0051 proliferates rapidly in red-fleshed pitaya juice, resulting in a good cholesterol-lowering ability of fermented juice (Maryati & Susilowati, 2018). Both *L. acidophilus* LA-05 and *Bifidobacterium lactis* BB-12 increased the bioaccessibility of some phenolics and enhanced the antioxidant activity of red-fleshed pitaya pulp (Morais et al., 2019). Thus, LAB fermentation may be a method to improve the quality of red-fleshed pitaya juice, although the specific impacts on its volatile and non-volatile compounds remain poorly characterized.

*L. paracasei* is a well-recognized safe probiotic and possesses strong carbohydrate and protein degradation capabilities, laying the foundation for its application in fruit juices. Previous studies have reported the viability of *L. paracasei* in red-fleshed pitaya juice, as well as its positive effects on the colour stability and anthocyanin composition (Usaga et al., 2022). We speculate that *L. paracasei* fermentation could improve the flavor and quality of red-fleshed pitaya juice. In this study, we determined the dynamic changes in the physicochemical properties, antioxidant activity, volatile and non-volatile metabolites of red-fleshed pitaya juice during *L. paracasei* fermentation. These results offer theoretical support to the biotransformation pathway of red-fleshed pitaya juice with *L. paracasei* fermentation.

## 2 Materials and methods

### 2.1 Materials and chemicals

Red-fleshed pitaya was purchased from a local market in Hefei (Anhui, China), while *L. paracasei* GBS04 was previously isolated from bamboo shoots and stored at −80 °C in our laboratory culture collection. de Man, Rogosa, and Sharpe (MRS) agar medium was prepared using basal components (peptone, beef extract, yeast extract, etc.) obtained from Sinopharm Chemical Reagents Co., Ltd. (Shanghai, China), following the standard formulation. We obtained 2,2-diphenyl-1-picryl-hydrazyl-hydrate (DPPH) and 2,2″-azino-bis-3-ethylbenzothiazoline-6-sulfonic acid (ABTS) from Yuanye Company Ltd. (Wuhan, China). Type II crude porcine pancreatic lipase and α-glucosidase were obtained from Aladin (Shanghai, China).

### 2.2 Fermentation of red-fleshed pitaya juice

We transferred *L. paracasei* to MRS plates and cultured them at 37 °C for 48 h in an incubator. The single colonies were inoculated into MRS broth medium at 37 °C for 24 h (OD_600_ = 1.4 ± 0.2). The cultures were centrifuged at 8000 ×*g* for 5 min to harvest the cells. The strains were then washed twice and resuspended in 0.85 % sterile saline (OD_600_ = 1.0). The red-fleshed pitaya fruit was washed with distilled water, peeled, diced, and then juiced in a juicer (JYL-C01S, Joyoung Co., Ltd., Jinan, China). The juice was filtered through gauze and pasteurized at 80 °C for 5 min in Erlenmeyer flasks (sealed with a cotton plug) to ensure microbial safety (SX-300, TOMY Company, Ltd., Japan), as described by Shi et al. (2023). After pasteurization, the inoculum (5 % *v*/v, 1.67 × 10^7^ CFU/mL) was added to the red-fleshed pitaya juice, followed by static incubation at 37 °C. The fermented juice was collected at 0, 2, 4, 6, 12, 24, and 36 h post-inoculation for subsequent analysis.

### 2.3 Analysis of physicochemical properties

#### 2.3.1 Determination of viable counts, pH, and total sugars

The viable counts of *L. paracasei* in red-fleshed pitaya juice at different fermentation times were determined on Plate Count Agar. The experimental steps for determining microbial viable count were performed as previously described by Shi et al. (2023). In brief, 1 mL of unfermented/fermented red-fleshed pitaya juice is added to 9 mL of sterile 0.9 % NaCl solution for gradient dilution. The appropriate dilution solution is poured onto MRS agar and incubated anaerobically at 37 °C for 48 h. The colonies were counted on plates yielding 30–300 colony-forming units (CFU), and microbial concentrations were recorded as CFU/mL. The pH of the red-fleshed pitaya juice was determined by a precision PHS-3C pH meter (Leici Instrument Co., Shanghai, China). Total sugar content was determined using the phenol sulfuric acid method and expressed as glucose equivalents (Shi et al., 2023). The experiments were performed with three independent technical replicates (*n* = 3).

#### 2.3.2 Total polyphenols, total flavonoids, and anthocyanin content

The total polyphenols, total flavonoids, and anthocyanin content in red-fleshed pitaya juice during fermentation were determined according to the experimental procedure of Shi et al. (2023). Total polyphenol (expressed as gallic acid equivalent) and flavonoid contents (expressed as rutin equivalent) were assayed using the Folin–Ciocalteu method and AlCl_3_ colorimetric method, respectively. The anthocyanin content is performed by mixing unfermented/fermented samples with acidified methanol and kept in a water bath at 60 °C for 1 h. The absorbance of the mixture is then measured at 657 nm, 620 nm, and 530 nm. The formula for calculating the anthocyanin content is detailed in the study by Shi et al. (2023). The experiments were performed with three independent technical replicates (*n* = 3).

### 2.4 Antioxidant activity and lipase and glucosidase inhibition

The ABTS-, DPPH-, and OH-scavenging abilities of red-fleshed pitaya juice at different time points during fermentation were detected and calculated as described by Shi et al. (2023). Briefly, the reaction mixture containing 1 mL ABTS^+^ working solution and 1 mL sample was incubated for 30 min before measuring absorbance at 734 nm. For the DPPH assay, 0.1 mL of sample was mixed with 3.9 mL of 0.025 g/L DPPH solution (39:1, *v*/v) and kept in the dark for 30 min, with absorbance measured at 517 nm. The OH- radical scavenging activity was assessed by incubating 1 mL of juice with 2 mL of 6 mM FeSO₄, 2 mL of 6 mM salicylic acid, and 2 mL of 6 mM H₂O₂, followed by 30 min of reaction before absorbance measurement at 510 nm. The experiments were performed with three independent technical replicates (*n* = 3).

The lipase and glucosidase inhibition abilities of red-fleshed pitaya juice at 0, 2, 4, 6, 12, 24, and 36 h of fermentation were determined according to the methods of Shi et al. (2023). The lipase inhibition assay was performed using *p*-nitrophenyl octanoate (NPC) and porcine pancreatic lipase (5 mg/mL). Reaction mixtures containing 100 μL samples and 100 μL enzyme solution were pre-incubated at 37 °C for 25 min, followed by addition of 100 μL NPC and further incubation (37 °C, 25 min). Absorbance was measured at 405 nm. The α-glucosidase inhibitory assay was performed by incubating 50 μL juice with 100 μL enzyme solution (0.5 U/mL, pH 6.8) in 96-well plates (37 °C, 10 min), followed by addition of 50 μL pNPG (5 mM) and absorbance measurement at 405 nm. Three biological and three technical replicates were analyzed for each sample (*n* = 3).

### 2.5 Volatile compounds

Volatile compounds in unfermented and fermented red-fleshed pitaya juice (six samples in each group, *n* = 6) were determined using headspace solid-phase microextraction gas chromatography–mass spectrometry (Thermo Fisher, CA, USA). 1 g of NaCl and 2-octanol (internal standard) were added to 5 mL sample and incubate at 60 °C for 10 min. The mixture was extracted using a 50/30 μm car/PDMS/DVB column for 30 min followed by transfer to a gas chromatograph injector and desorbed at 250 °C for 5 min. Analysis was performed using GC–MS combined with a TR-5MS column and a quadrupole DSQ II MS. The thermal program was initiated at 50 °C for 3 min, followed by a linear temperature ramp of 5 °C/min to reach 240 °C, which was maintained for 10 min. Mass spectrometric analysis was performed in electron impact ionization mode with an ionization energy of 70 eV, employing full scan acquisition over a mass range of 45–400 amu. Compounds were identified by mass spectrometry comparison with the NIST11 mass spectrometry database and Wiley. The abundance of compounds was determined by peak area and normalized according to internal standards.

### 2.6 Untargeted profiling of non-volatile compounds

Non-volatile compounds in unfermented and fermented red-fleshed pitaya juice (six samples in each group, *n* = 6) were determined using ultra-high performance liquid chromatography (Thermo Fisher Scientific, MA, USA) with quadrupole time-of-flight mass spectrometry. Samples were centrifuged (8000 ×*g*, 10 min), and the supernatants were extracted with 80 % methanol under ultrasonication (40 min, 4 °C, 80 kHz). The supernatants after centrifugation (12,000 ×*g*, 10 min) were freeze-dried and reconstituted in 80 % methanol for UPLC-QTOF-MS analysis. Metabolite separation was achieved using a Waters Acquity UPLC HSS T3 reversed-phase column (2.1 × 100 mm, 1.8 μm) maintained at 35 °C. Mass spectrometric analysis was performed using a heated electrospray ionization source operating in both positive and negative ion modes. The mobile phases consist of 0.1 % formic acid in water (A) and methanol (B) in positive ion mode, and water (A) and pure methanol (B) in negative ion mode, with a flow rate of 0.3 mL min^−1^. The mobile phase gradient was as follows: 0–1.0 min (2 %, B); 1.0–10 min (2–98 %, B); 10–12 min (98 %, B); and 12–15 min (2 %, B). Mass spectrometry conditions included ESI voltages of 3.8 kV (positive) and 2.8 kV (negative). The MS1 acquisition was performed with a scan range of *m*/*z* 50–1070 at 70,000 resolution, using an automatic gain control target of 1 × 10^6^ and a maximum ion injection time of 100 ms, while MS2 analysis was conducted at 17,500 resolution with normalized collision energies of 20, 40, and 60 eV, an AGC target of 5 × 10^4^, a maximum injection time of 50 ms, a dynamic exclusion duration of 10 s, and fragmentation of the top 7 most intense ions.

### 2.7 Sensory evaluation analysis

The sensory characteristics of unfermented and fermented red-fleshed pitaya juice were evaluated by 10 untrained panelists. The following sensory attributes were assessed on a 9-point scale: appearance, taste, flavor, viscosity, and palatability. The sensory evaluation scores were plotted on a radar chart for better visual display.

### 2.8 Statistical analysis

Principal component analysis (PCA), partial least squares discriminant analysis (PLS-DA), and orthogonal partial least squares discriminant analysis (OPLS-DA) were performed using the online platform of MetaboAnalyst 6.0 (http://www.metaboanalyst.ca) and Origin 2021 software. Metabolite identification and quantification were carried out using Compound Discoverer software (v3.8). Metabolic pathway analysis was subsequently performed using the MetaboAnalyst 6.0 platform. The results are presented as mean ± standard error. Statistical significance was assessed using one-way analysis of variance (ANOVA) followed by Tukey's post hoc test for multiple comparisons (*p* < 0.05), and the differences between two groups were assessed using a *t*-test (SPSS 16.0), with *p* < 0.05 considered statistically significant.

## 3 Results and discussion

### 3.1 Viable cell number, pH value, and total sugars during fermentation

**Fig. 1:**
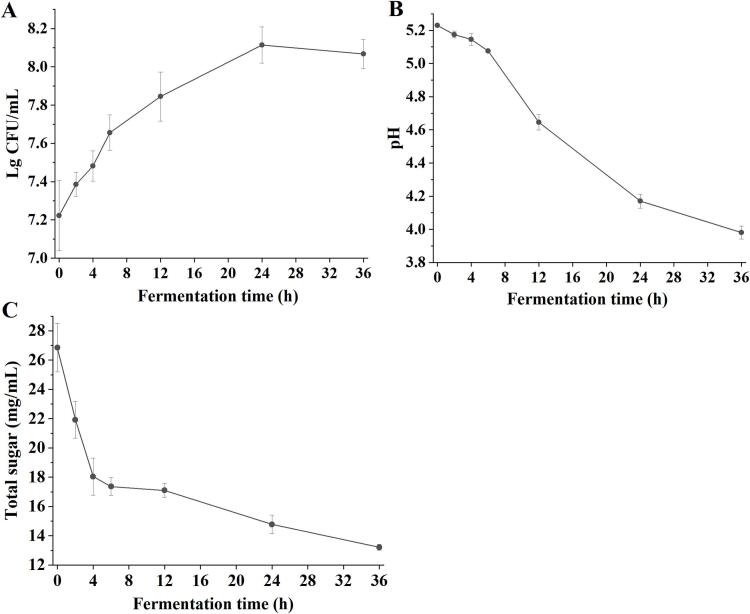
Viable cell number (A), pH value (B), and total sugars (C) of red-fleshed pitaya juice during *Lacticaseibacillus paracasei* fermentation. (For interpretation of the references to colour in this figure legend, the reader is referred to the web version of this article.)

Red-fleshed pitaya juice was initially inoculated with 1.67 × 10^7^ CFU/mL of *L. paracasei* (Fig. 1A). During fermentation, the viable count of *L. paracasei* significantly increased, reaching a maximum of 1.30 × 10^8^ CFU/mL at 24 h, and remained constant until 36 h (*p < 0.05*). The observed viable count is consistent with previous reports in similar substrates, where Morais et al. (2019) reported *L.* *acidophilus* LA-05 reaching 8.59 ± 0.18 log CFU/mL (equivalent to ∼3.89 × 10^8^ CFU/mL) in red pitaya pulp after 48 h fermentation. The pH of red-fleshed pitaya juice was 5.23 ± 0.01, which decreased to 3.98 ± 0.29 after 36 h of fermentation (Fig. 1B). It was previously reported that the pH of fresh dragon fruit juice decreased from 5.61 to 3.49 after 48 h of fermentation, which was similar with our result (Muhialdin et al., 2020). Sugar is an important carbon source for lactic acid bacteria in fermented red-fleshed pitaya juice. The trend of total sugars was consistent with that of the viable number of bacteria and pH, significantly decreasing from 26.20 ± 1.66 mg/mL to 13.24 ± 0.20 mg/mL (Fig. 1C). These results suggested that *L. paracasei* is generally well adapted to the pitaya juice substrate.

### 3.2 Total polyphenols, total flavonoids, and anthocyanin concentration during fermentation

Red-fleshed pitaya contains abundant phytochemicals, such as polyphenols, flavonoids, and anthocyanins, along with vitamins and minerals. It is rich in diverse polyphenolic compounds, with the major compounds identified as: chlorogenic acid (19.14–19.45 mg/kg dry weight), ferulic acid (10.72–25.04 mg/kg), p-coumaric acid (8.62–16.29 mg/kg) and caffeic acid (5.44–57.03 mg/kg) (Tang et al., 2021; Tenore et al., 2012). Numerous lactic acid bacteria have demonstrated strong polyphenol hydrolase activity for the hydrolysis of large polyphenols into polyphenols with small molecular masses and unconjugated glycosides, thus exhibiting enhanced physiological activity (Yang et al., 2023). Previous studies have reported the positive effects of lactic acid bacteria fermentation on juice quality. In particular, *L. acidophilus* LA-05 and *Bifidobacterium animalis* subsp. *lactis* BB-12 fermentation increased the total phenolic content of red pitaya pulp and the bioaccessibility of catechin, epigallocatechin gallate, and procyanidin B2 (Morais et al., 2019).

**Fig. 2:**
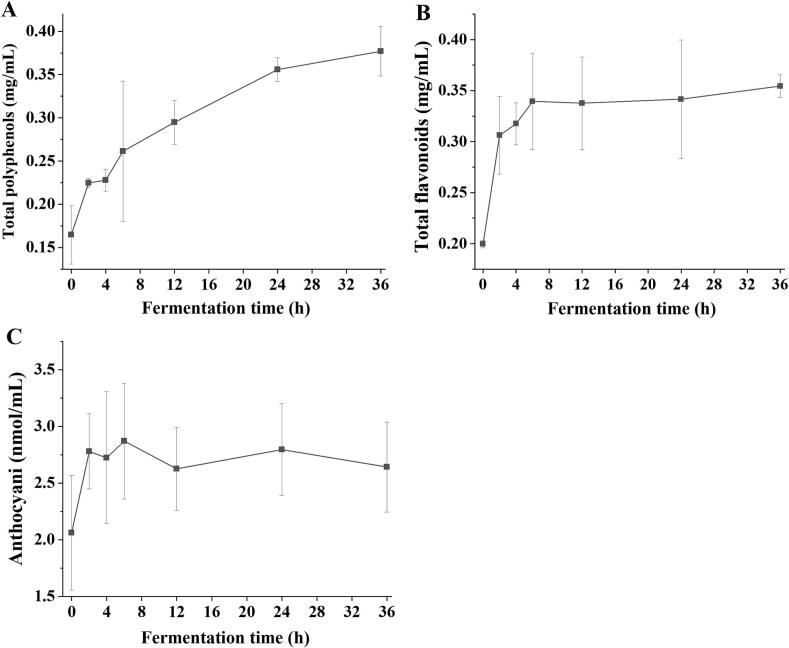
Total polyphenol (A), total flavonoid (B), and anthocyanin (C) contents of red-fleshed pitaya juice during *L. paracasei* fermentation. (For interpretation of the references to colour in this figure legend, the reader is referred to the web version of this article.)

In this study, *L. paracasei* significantly increased the total polyphenol content of red-fleshed pitaya juice from 0.16 ± 0.03 mg/mL to 0.38 ± 0.03 mg/mL (Fig. 2A). The growth and metabolic activity of *L. paracasei* can catalyze the biotransformation of polyphenolic compounds, converting them into bioactive derivatives with potentially enhanced physiological effects (Yang et al., 2023). The content of total flavonoids in red-fleshed pitaya juice was 0.20 ± 0.00 mg/mL, which was rapidly increased by *L. paracasei*, reaching a maximum of 0.34 ± 0.05 mg/mL within 6 h and remaining stable during fermentation (Fig. 2B). However, previous studies involving *Lactobacillus* fermentation have reported either increased or decreased levels of total polyphenols and flavonoids. Shi et al. (2024) observed that *L. paracasei* fermentation significantly reduced its flavonoid content by 7.35 %. In contrast, *L. paracasei* fermentation significantly increased the total flavonoid content of *Rosa roxburghii* Tratt juice (Luo et al., 2024). This inconsistency arises from the microbial fermentation-induced metabolism of flavonoid compounds, including the degradation or polymerization of flavonoids into dihydroxy flavonoid analogues, as well as alterations in enzyme activity, such as glycosidase, glycosyltransferase, and tannase. Although the total flavonoid content may decrease, the resulting structural modifications can substantially enhance their biological activity (He et al., 2022).

Anthocyanins are important fruit pigments that partially contribute to colour formation and are abundantly available in the pulp and peel of red-fleshed pitaya. Although anthocyanins are not the main phytochemicals present in red pitaya fruits, they have multiple health benefits (Khoo et al., 2022). The total anthocyanin content of fresh red pitaya pulp was 159.7 ± 8.90 mg/g (dried sample) (Saenjum et al., 2021). In our study, the anthocyanin content of unfermented red-fleshed pitaya juice was 2.06 ± 0.50 nmol/mL (Fig. 2C). The anthocyanin content of the fermented juice increased significantly to 2.87 ± 0.50 nmol/mL after 6 h and showed no significant variation during 30 h of fermentation, demonstrating stability throughout the fermentation period. This acidic environment helps preserve anthocyanin structure by maintaining the flavylium cation form (Enaru et al., 2021). Therefore, *L. paracasei* fermentation significantly increased the contents of polyphenols, flavonoids, and anthocyanins in red-fleshed pitaya juice, thereby contributing to its potential as a functional food.

### 3.3 Antioxidant activity

**Fig. 3:**
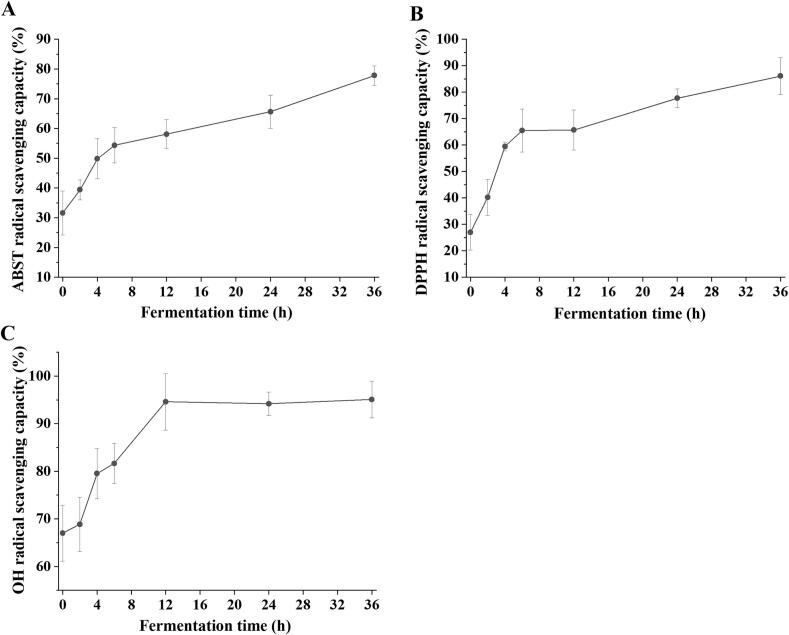
ABTS- (A), DPPH- (B), and OH- (C) scavenging activities of red-fleshed pitaya juice before and after *L. paracasei* fermentation. (For interpretation of the references to colour in this figure legend, the reader is referred to the web version of this article.)

The ABTS-, DPPH-, and OH-scavenging activities of red-fleshed pitaya juice were measured to assess its antioxidant capacity (Fig. 3). The ABTS^+^-scavenging activity of the fermented red-fleshed pitaya juice increased rapidly to 58.10 % at 6 h and significantly increased to a maximum of 77.79 % at 36 h. The DPPH^+^-scavenging ability showed a similar trend, reaching a maximum of 86.08 % after 36 h of fermentation, while the OH^−^-scavenging ability increased significantly to 94.59 % after 12 h of fermentation. Therefore, *L. paracasei* fermentation significantly improved the antioxidant capacity of red-fleshed pitaya juice.

Red-fleshed pitaya juice is a good source of antioxidants (Tang et al., 2021; Tenore et al., 2012). Fermentation improves antioxidant activity mainly because of an increase in the amount of phenolic compounds and flavonoids during fermentation, which is a result of microbial hydrolysis reactions. Lactic acid bacteria can de-carboxylate, de-esterify, de-methylate, and de-glycosylate polyphenols, resulting in their biotransformation into compounds with high bioavailability and bioactivity (Hur et al., 2014). Previous studies have reported that fermentation with lactic acid bacteria, such as *L.* *plantarum*, *L. mesenteroides*, and *Lactobacillus brevis*, increased the antioxidant properties of fruit juices (Ávila et al., 2009). *L. plantarum* ATCC14917 fermentation improved the DPPH- and ABTS-scavenging capacity and cellular antioxidant capacity of apple juice, primarily through increased 5-O-caffeoylquinic acid, quercetin, and phloretin levels (Li et al., 2018).

### 3.4 Lipase and α-glucosidase inhibition activities

**Fig. 4:**
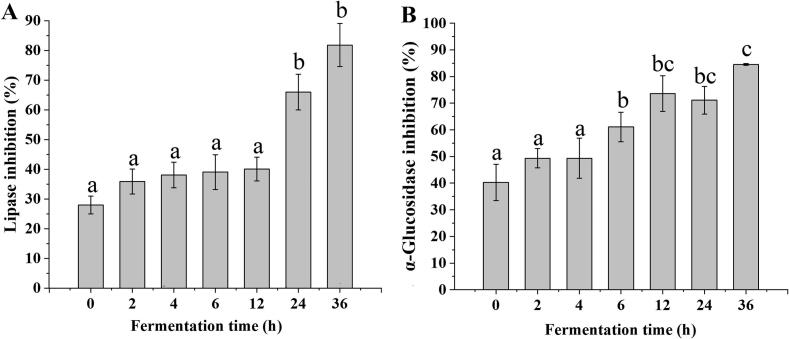
Lipase (A) and α-glucosidase (B) inhibition activities of red-fleshed pitaya juice before and after *L. paracasei* fermentation. (For interpretation of the references to colour in this figure legend, the reader is referred to the web version of this article.)

The α-glucosidase enzyme lowers blood glucose levels by reducing intestinal absorption of carbohydrates, and pancreatic lipase regulates the digestion and absorption of dietary lipids (Hong et al., 2021). Lipase and α-glucosidase inhibition activities of red-fleshed pitaya juice were investigated during fermentation. The pancreatic lipase inhibition activity of unfermented red-fleshed pitaya juice was 27.95 %, which significantly increased to 65.99 % after 24 h of fermentation with *L. paracasei* and reached a maximum of 81.82 % after 36 h of fermentation (Fig. 4A). Meanwhile, the α-glucosidase inhibition activity of red-fleshed pitaya juice significantly increased after 6 h of fermentation (61.06 %) and increased significantly to 84.54 % after 36 h of fermentation (Fig. 4B).

These results indicate that *L. paracasei* fermentation significantly increased the lipase and α-glucosidase inhibition ability of red-fleshed pitaya juice, thus contributing to reduced absorption of carbohydrates and fats in the diet. Zhang et al. (2021) reported that *L.* *plantarum* improved the α-glucosidase and lipase inhibition activities of blueberry juice. Yao et al. (2024) reported 38.72 % inhibition of lipase by fig juice fermented with lactic acid bacteria. The inhibitory effect on lipase and α-glucosidase activities may be related to the antioxidant capacity attributed to increased bioactive compounds, such as polyphenols and flavonoids. Polyphenols and flavonoids can be embedded in the catalytic cavity and interact with key amino acids in the catalytic triad, resulting in enzyme inhibition (Li et al., 2023). This mechanism aligns with Zhang et al.'s findings that quercetin and catechins competitively bind to α-glucosidase and pancreatic lipase active sites, forming hydrogen bonds and hydrophobic interactions with residues (Zhang et al., 2017).

### 3.5 Volatile compounds

**Fig. 5:**
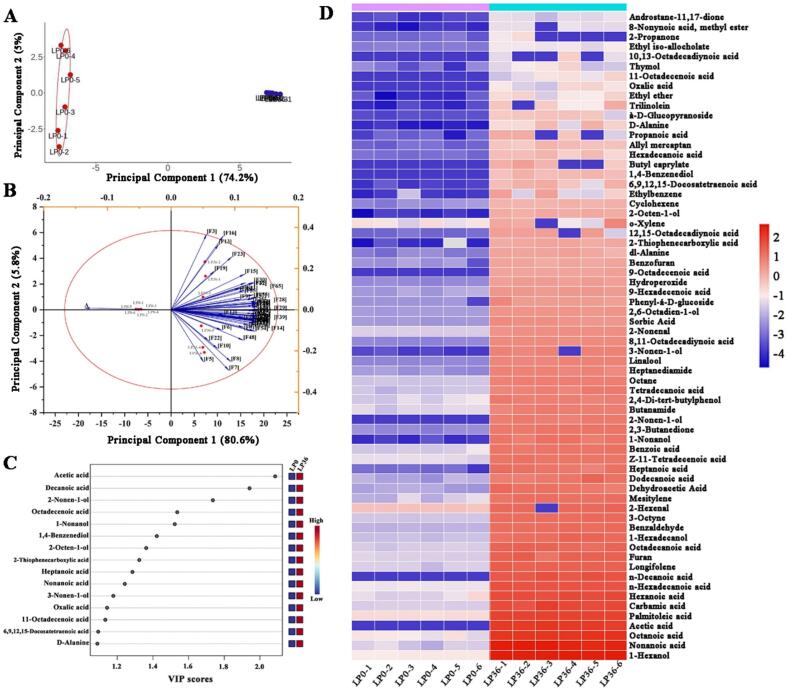
Volatile compounds profiling of red-fleshed pitaya juice before (LP0) and after (LP36) *L. paracasei* fermentation. (A and B) Principal component analysis (PCA) showing (A) sample clustering and (B) compound loadings. (C) Variable importance in projection (VIP) scores from PLS-DA model. (D) Heatmap of differential volatiles (*p < 0.05*, *n* = 6). (For interpretation of the references to colour in this figure legend, the reader is referred to the web version of this article.)

Volatile compounds are important indicators of fruit quality and flavor, and play an important role in determining consumer acceptance of a product. A total of 78 volatile compounds were detected in fermented (LP0) and unfermented (LP36) red-fleshed pitaya juice, including acids (36), alcohols (10), aldehydes (7), esters (5), and ketones (5) (Fig. 5). Aldehydes were the most abundant volatile compounds in unfermented red-fleshed pitaya juice, with 2-hexenal showing the highest relative abundance of 31.05 %. Notably, 2-hexenal provides a “grassy” note and is one of the main components in the aroma of Honeycrisp apples (Wu et al., 2022). Another major compound was palmitoleic acid (C16:1), which is an omega-7 monounsaturated fatty acid that is abundant in plants and has been reported to alleviate metabolic abnormalities.

To identify the key volatile compounds, we performed relative odor activity value (ROAV) analysis on the differential compounds. Compounds with ROAV>1 were identified as major contributors to the overall aroma profile. As presented in Table 1, we detected 18 volatile compounds with ROAV>1, indicating their potential sensory significance. Furthermore, PCA and PLS-DA were performed to differentiate the key volatile flavor substances between unfermented and fermented red-fleshed pitaya juice samples. (Fig. 5A and B). According to the variable importance in projection (VIP) scores, the key volatile flavor substances were identified after fermentation, among which nine were acids (Fig. 5C). The acid content in red-fleshed pitaya juice increased significantly after 36 h of *L. paracasei* fermentation, with six acids representing 52.01–59.87 % of the total volatile substances. The accumulation of organic acid aligns with the observed pH decline (from 5.23 ± 0.01 to 3.98 ± 0.29). Acetic acid is one of the main products of lactic acid bacterial metabolism, and *L. paracasei* increased the acetic acid content in red-fleshed pitaya juice to 149.97 ± 16.71 μg/mL. Other organic acids, such as decanoic acid, nonanoic acid, and hexanoic acid, also provide rich flavors and may impart fatty, cheesy, and sour flavors (ROAV>1). Octanoic acid (ROAV>1) was one of the most abundant acids, contributing butter and almond aromas. Moreover, a significant increase in acetic, dodecanoic, and hexanoic acids implied that *L. paracasei* fermentation contributed to fatty acid formation in red-fleshed pitaya juice (Li et al., 2024).

**Table 1:** Key volatile compounds (ROAV>1, VIP > 1) identified in red-fleshed pitaya juice before and after L. *paracasei* fermentation.

Volatile compounds	Odor descriptor	LP0 (ug/mL)	LP36 (ug/mL)	Odor Threshold (ug/mL)
**Acids**				
Nonanoic acid	Fatty, coconut	0.03 ± 0.01	424.20 ± 36.98	3.9 × 10^−3^
Octanoic acid	Fruity-acid	0.10 ± 0.05	186.78 ± 6.66	8.0 × 10^−3^
Acetic acid	Sour, vinegar-like	ND	149.94 ± 16.71	0.03 × 10^−2^-0.15
Hexanoic acid	Goat-like	0.07 ± 0.01	73.03 ± 8.25	0.3
n-Hexadecanoic acid	Virtually odorless	0.06 ± 0.00	55.37 ± 4.91	1
n-Decanoic acid	Unpleasant	ND	48.56 ± 9.40	0.22–102
Octadecanoic acid	Tallow	0.02 ± 0.00	24.09 ± 3.80	20
Heptanoic acid	Tallow-like	<0.01	13.32 ± 3.69	10.4–640
Propanoic acid	Slightly pungent	<0.01	0.13 ± 0.01	0.02–0.17
2-Thiophenecarboxylic acid	N/A	<0.01	1.59 ± 1.25	N/A
Oxalic acid	Odorless	ND	0.12 ± 0.01	N/A
11-Octadecenoic acid	N/A	ND	0.07 ± 0.04	N/A
6,9,12,15-Docosatetraenoic acid	N/A	<0.01	0.62 ± 0.29	N/A
**Alcohols**				
1-Hexanol	Sweet alcohol, pleasant	0.11 ± 0.01	452.23 ± 26.71	0.007–0.01
1-Hexadecanol	Faint odor	0.02 ± 0.00	20.46 ± 3.74	N/A
1-Nonanol	Floral odor	<0.01	12.08 ± 2.86	0.01
Linalool	Floral odor	<0.01	7.15 ± 0.58	1
2-Nonenal	Fatty, green	0.03 ± 0.00	4.36 ± 0.28	2.5 × 10^−4^
**Aldehydes**				
2-Hexenal	Honeycrisp apples	0.52 ± 0.02	18.73 ± 1.26	17
**Others**				
Cyclohexene	Sweet odor	<0.01	0.96 ± 0.07	0.18
Furan	Nutty aroma	0.03 ± 0.00	28.32 ± 2.08	N/A
Mesitylene	Aromatic odor	0.02 ± 0.00	16.31 ± 2.03	2
Ethylbenzene	Aromatic odor	<0.01	0.82 ± 0.13	0.09–0.6
2-Nonen-1-ol	Fruity, green	ND	9.41 ± 0.82	N/A
1,4-Benzenediol	Odorless	<0.01	0.80 ± 0.19	N/A
2-Octen-1-ol	N/A	<0.01	1.34 ± 0.17	N/A
3-Nonen-1-ol	N/A	<0.01	5.97 ± 2.69	N/A
D-Alanine	N/A	<0.01	0.42 ± 0.41	N/A
Thymol	Thyme, aromatic odor	<0.01	0.09 ± 0.01	0.001–0.01

ND: not detected. N/A: not available.

Odor thresholds and descriptors provenance are provided by Leibniz-LSB@TUM Odorant Database and PubChem (https://pubchem.ncbi.nlm.nih.gov).

Alcohols are essential compounds produced through microbial fermentation. Alcohols not only provide an aroma to the juice but also act as solvents for other aromatic compounds, thus contributing to the retention of the aroma. Overall, 1-hexanol (ROAV>1) was the most significantly increased component in fermented red-fleshed pitaya juice, reaching 452.23 μg/mL and providing a marzipan-like flavor (Fig. 5D). It is produced via the enzymatic oxidation of the fatty acid linoleic acid. A previous study reported a significant increase in 1-hexanol to 566.3 μg/mL after the fermentation of Chinese wolfberry juice with *L. paracasei* (Qi et al., 2021). 2-nonen-1-ol is considered the major volatile component in watermelon juice and was the key volatile component in fermented red-fleshed pitaya juice (Mandha et al., 2021). Additionally, 1-nonanol (fresh, orange, and pink notes) was a significant contributor to volatile flavors after fermentation (ROAV>1), adding floral and fruity notes to red-fleshed pitaya juice (Wang et al., 2018).

In addition to the acids and alcohols that contributed significantly to the flavor of fermented red-fleshed pitaya juice, other aldehydes, terpenes, and amino acids were significantly increased (Fig. 5D). Terpenoids, such as longifolene, are widely found in plants and are less abundant in fruit juices; however, because of their generally low threshold, they give juices fruity and sweet flavors. A significant increase in benzaldehyde was also observed in the fermented red-fleshed pitaya juice, imparting a pleasant sweet aroma and enhancing its organoleptic qualities. Additionally, d-alanine, a sweet-tasting amino acid that contributes significantly to the sweetness of fermented red-fleshed pitaya juice, was observed after fermentation. These results suggest that *L. paracasei* fermentation increased the variety and content of volatile compounds, which added floral, fruity, buttery, sour, and sweet flavors to improve the flavor of red-fleshed pitaya juice.

### 3.6 Non-volatile compounds

**Fig. 6:**
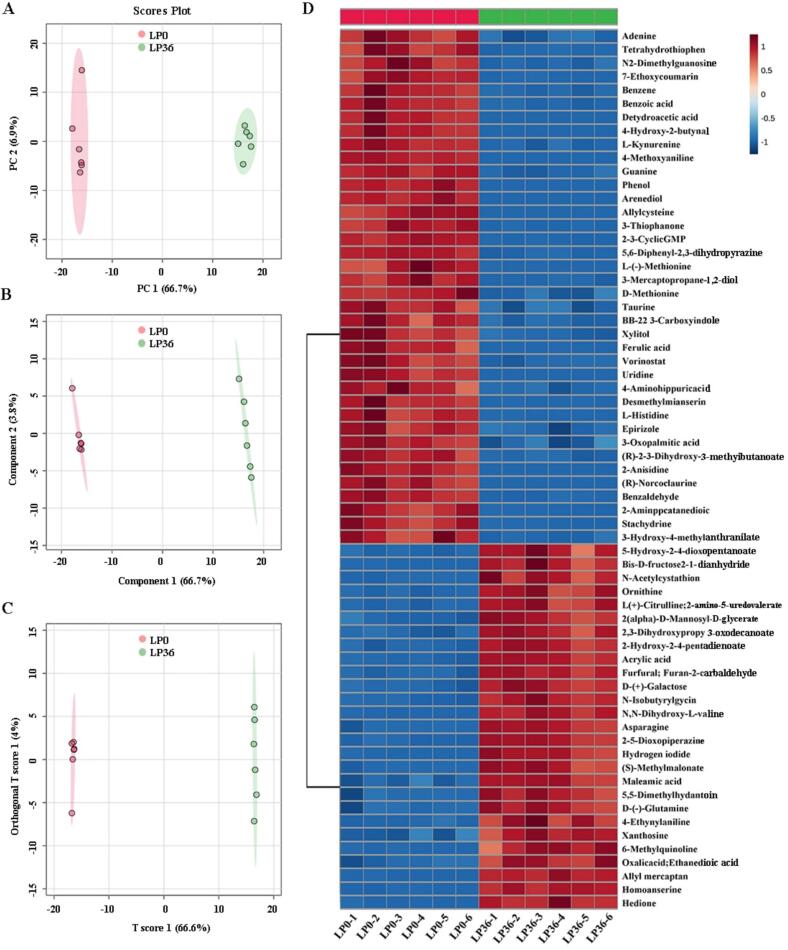
Non-volatile compounds in red-fleshed pitaya juice before (LP0) and after (LP36) *L. paracasei* fermentation. (A) PCA score plot. (B) PLS-DA score plot. (C) OPLS-DA score plot. (D) Heatmap of differential non-volatile compounds (*p < 0.05*, n = 6). (For interpretation of the references to colour in this figure legend, the reader is referred to the web version of this article.)

**Fig. 7:**
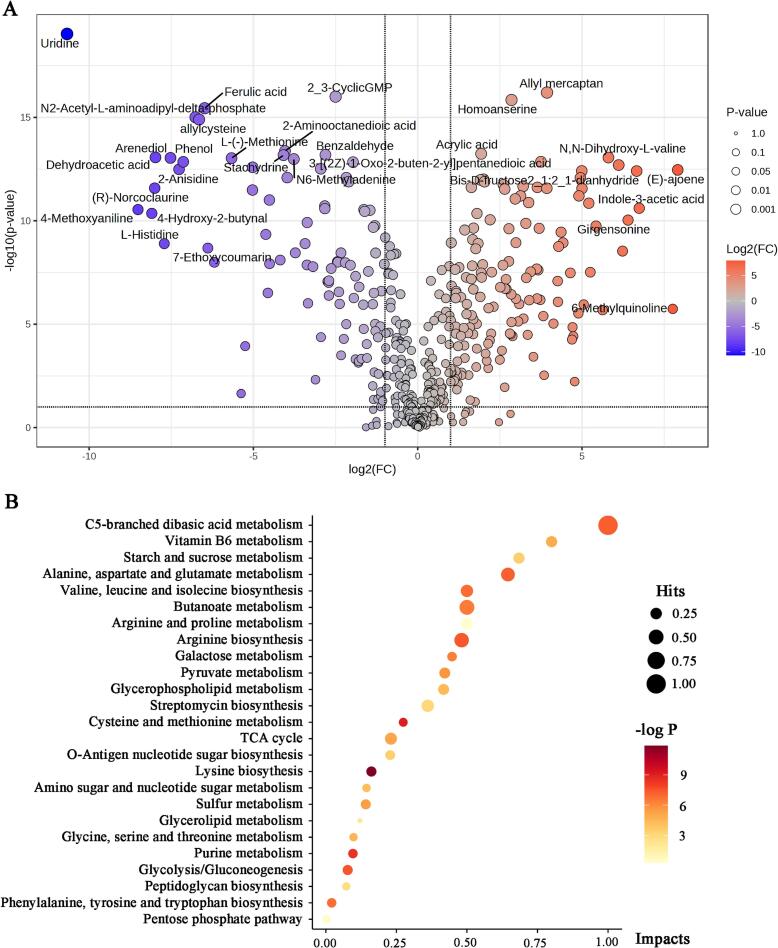
(A) Volcanic plot of red-fleshed pitaya juice sample distribution before and after *L. paracasei* fermentation. (B) Metabolite pathway analysis based on KEGG enrichment analysis. (For interpretation of the references to colour in this figure legend, the reader is referred to the web version of this article.)

Individual lactic acid bacterial fermentations produce unique metabolic profiles that affect the quality, characteristics, and functions of fermentation products. Untargeted metabolomics was used to determine the content of non-volatile compounds in red-fleshed pitaya juice before and after fermentation (Fig. 6, Fig. 7). Overall, 448 metabolites were identified, of which 146 significantly increased and 95 significantly decreased. The PCA results showed that the post-fermentation red-fleshed pitaya juice samples were discrete, whereas the pre-fermentation samples were aggregated. Furthermore, the post-fermentation red-fleshed pitaya juice samples were completely separated from the pre-fermentation samples. The results of PCA, PLS-DA, and OPLS-DA indicated that *L. paracasei* fermentation influenced the non-volatile components of red-fleshed pitaya (Fig. 6A, B, and C).

In particular, *L. paracasei* fermentation resulted in the most significant decrease in uridine content in red-fleshed pitaya juice (Figs. 6D and 7A). This result is consistent with that of a previous study by Zhao et al. (2024), wherein the significant decrease in the uridine content in *Lactobacillus*-fermented tomato juice was due to the utilization of uridine as a carbon source by *Lactobacillus* (Zhao et al., 2024). Uridine is involved in pyrimidine metabolism during *L. paracasei* fermentation. In addition, the ferulic acid content was significantly reduced in fermented red-fleshed pitaya juice. Ferulic acid is a phenolic compound commonly found in plants that has been shown to be significantly reduced in apple and blueberry juices fermented using a variety of lactic acid bacteria (Wu et al., 2020).

The substance that increased most after the fermentation of red-fleshed pitaya juice was allyl mercaptan. In a previous study, garlic and onions fermented using *Lactobacillus pentosus* SMB718 showed higher levels of allyl mercaptan, which has a favorable aroma and multiple health benefits. Several studies have shown that allyl mercaptan reduces cholesterol synthesis (Jo et al., 2020). The significant increases in ornithine, asparagine, and glutamine levels also indicated that fermentation with *L. paracasei* improved the nutritional profile of red-fleshed pitaya juice. The significant increase in ornithine content may be due to the conversion of arginine to ornithine by arginine deiminase (Vrancken et al., 2009). Notably, the content of indole-3-lactic acid (alkaloid), which is a well-known compound involved in tryptophan metabolism and has antioxidant and immunomodulatory properties, was also significantly increased in fermented red-fleshed pitaya juice. These results suggest that red-fleshed pitaya juice fermented with *L. paracasei* can serve as a potential functional food.

Pathway analysis revealed major enrichment of differentially expressed metabolites after the fermentation of red-fleshed pitaya juice with *L. paracasei* (Fig. 7B). Six pathways that play important roles in the overall metabolic network were identified and are mainly related to carbohydrate metabolism, amino acid synthesis and metabolism, and lipid metabolism. In particular, the C5-branched dibasic acid metabolic pathway has an essential influence on the metabolic processes of organisms and is associated with carbohydrate metabolism and energy production. Other pathways associated with carbohydrate metabolism with the highest abundance were starch and sucrose metabolism, butanoate metabolism, galactose metabolism, pyruvate metabolism, tricarboxylic acid cycle, glycolysis/gluconeogenesis and pentose phosphate.

Vitamin B6 metabolism pathway also played important role in the overall metabolic network. Vitamin B6 is a component of coenzymes involved in a variety of metabolic reactions and is closely related to the metabolism of amino acids. In this study, we identified eight pathways related to amino acid synthesis and metabolism. Alanine, aspartate, and glutamate metabolic are critical metabolic pathways involved in fermentation. They are involved in protecting lactic acid bacteria against damage caused by low-pH environments. The significant increase in glutamate content after fermentation enhanced the umami flavor of the juice. Additionally, valine, leucine, and isoleucine share a metabolic pathway, with valine being the main component significantly increased during *L. paracasei* fermentation. These branched-chain amino acids are essential amino acids that are critical for protein synthesis, energy production, and various metabolic processes in living organisms. These results further confirm that *L. paracasei* fermentation improved the nutritional composition of red-fleshed pitaya juice. Furthermore, amino acids can exhibit sour, sweet, and buttery characteristics and play an important role in the flavor profile of plant fermentation products.

### 3.7 Sensory evaluation

**Fig. 8:**
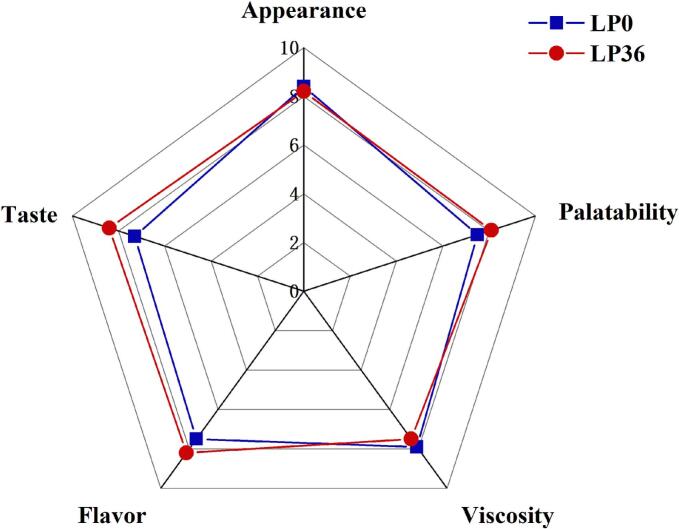
Sensory evaluation of the unfermented (LP0)/fermented (LP36) red-fleshed pitaya juice by *L. paracasei*. (For interpretation of the references to colour in this figure legend, the reader is referred to the web version of this article.)

Further comparisons were performed between the sensory scores of unfermented and fermented red-fleshed pitaya juice (Fig. 8). The appearance and viscosity of the fermented juice were statistically similar to those of the unfermented juice. Meanwhile, the texture, flavor, and palatability of the fermented juice were significantly higher than those of the unfermented juice. These results indicated that *L. paracasei* GBS04 fermentation not only preserved the visual and texture of red-fleshed pitaya juice, but also improved its sensory appeal, thereby being more attractive for consumers.

## 4 Conclusion

In summary, *L. paracasei* fermentation increased the total polyphenol (2.29-fold increase), flavonoid (1.77-fold increase), and anthocyanin (1.28-fold increase) contents of red-fleshed pitaya juice. The antioxidant capacity and lipase and α-glycosidase inhibition activity of fermented red-fleshed pitaya juice were improved. Furthermore, *L. paracasei* fermentation increased the abundance of volatile metabolites. The elevated levels of acetic acid, 1-hexanol, and 1-nonanol imparted flavor characteristics, including floral, fruity, sour, and sweet notes. Additionally, *L. paracasei* fermentation improved the non-volatile components of red-fleshed pitaya juice, particularly ornithine, asparagine, glutamine, and indole-3-lactic acid, suggesting potential nutritional modulation. The results indicate that *L. paracasei* fermentation could improve physicochemical properties, the antioxidant activity, volatile metabolites, and non-volatile metabolites of red-fleshed pitaya juice. Therefore, *L. paracasei* shows potential as a fermentation strain for red-fleshed pitaya juice. However, the specific functional components in red-fleshed pitaya, such as betalains, remain uncharacterized in this study. Future work will quantify betalain content (e.g., via HPLC-DAD) and evaluate their contribution to antioxidant and enzyme inhibitory effects through purified compound assays.

## CRediT authorship contribution statement

**Jiayu Gu:** Writing – original draft, Investigation, Data curation. **Qian Zhu:** Writing – review & editing, Conceptualization. **Jingjing Du:** Writing – review & editing, Methodology. **Jiagang Guo:** Conceptualization. **Yuhan Wu:** Supervision, Resources, Methodology. **Song Yang:** Supervision, Resources, Project administration. **Jian Jiang:** Supervision, Conceptualization.

## Declaration of competing interest

The authors declare that they have no known competing financial interests or personal relationships that could have appeared to influence the work reported in this paper.

## Data Availability

Data will be made available on request.
